# Rapid activation of esophageal mechanoreceptors alters the pharyngeal phase of swallow: Evidence for inspiratory activity during swallow

**DOI:** 10.1371/journal.pone.0248994

**Published:** 2021-04-02

**Authors:** Michael L. Frazure, Alyssa D. Brown, Clinton L. Greene, Kimberly E. Iceman, Teresa Pitts

**Affiliations:** 1 Department of Neurological Surgery and Kentucky Spinal Cord Injury Research Center, College of Medicine, University of Louisville, Louisville, Kentucky, United States of America; 2 Department of Physiology, University of Louisville, Louisville, Kentucky, United States of America; 3 School of Medicine, University of Louisville, Louisville, Kentucky, United States of America; 4 Department of Physiology and Biomedical Engineering, Mayo Clinic College of Medicine, Rochester, Minnesota, United States of America; National Yang-Ming University, TAIWAN

## Abstract

Swallow is a complex behavior that consists of three coordinated phases: oral, pharyngeal, and esophageal. Esophageal distension (EDist) has been shown to elicit pharyngeal swallow, but the physiologic characteristics of EDist-induced pharyngeal swallow have not been specifically described. We examined the effect of rapid EDist on oropharyngeal swallow, with and without an oral water stimulus, in spontaneously breathing, sodium pentobarbital anesthetized cats (*n* = 5). Electromyograms (EMGs) of activity of 8 muscles were used to evaluate swallow: mylohyoid (MyHy), geniohyoid (GeHy), thyrohyoid (ThHy), thyropharyngeus (ThPh), thyroarytenoid (ThAr), cricopharyngeus (upper esophageal sphincter: UES), parasternal (PS), and costal diaphragm (Dia). Swallow was defined as quiescence of the UES with overlapping upper airway activity, and it was analyzed across three stimulus conditions: 1) oropharyngeal water infusion only, 2) rapid esophageal distension (EDist) only, and 3) combined stimuli. Results show a significant effect of stimulus condition on swallow EMG amplitude of the mylohyoid, geniohyoid, thyroarytenoid, diaphragm, and UES muscles. Collectively, we found that, compared to rapid cervical esophageal distension alone, the stimulus condition of rapid distension combined with water infusion is correlated with increased laryngeal adductor and diaphragm swallow-related EMG activity (schluckatmung), and post-swallow UES recruitment. We hypothesize that these effects of upper esophageal distension activate the brainstem swallow network, and function to protect the airway through initiation and/or modulation of a pharyngeal swallow response.

## 1. Introduction

Swallow is an important, complex behavior, controlled by a pattern generator in the medulla [1–3]. A robust swallow pattern consists of three coordinated phases that propel the bolus in a rostral to caudal direction: oral, pharyngeal and esophageal [1, 4–9]. The pharyngeal phase of swallow is characterized by hyolaryngeal elevation, laryngeal adduction, and pharyngeal constriction, with concurrent relaxation of the upper esophageal sphincter (UES) and activation of inspiratory muscles (i.e. schluckatmung, or “swallow breath”); the pattern of muscle activation is rapid and stereotypic [10–12]. The sequential activation of the muscles involved in swallow is tightly coordinated to regulate pressures in the thoracic cavity and upper airway [13–15]. These pressures must be highly regulated to control the passage of a bolus into the esophagus or air into the lungs via a dual valve system [16]. In order for a bolus to enter the esophagus, the UES must relax, and the tongue and pharyngeal muscles activate to propel the bolus. This is aided by the diaphragm, such that negative intra-thoracic pressure paired with positive pressure in the oropharynx produces a pressure differential to optimize proper bolus movement into the esophagus. This must be accomplished while avoiding aspiration into the airway [16–19].

The oropharyngeal phase of swallow strongly influences the esophageal phase, either via direct excitation/disinhibition, by more diffuse neuromodulation, and/or afferent feedback [20–25]. These afferents include oropharyngeal receptors, laryngeal/thoracic receptors, pulmonary stretch receptors, esophageal stretch receptors, and possibly thoracic-abdominal receptors (traveling through spinal dorsal root ganglia) [6, 7, 17, 26–38]. Motor contraction during swallow must adapt to the size of the bolus, based on afferent peripheral feedback. Distension of the pharynx by a bolus modulates both the oropharyngeal and esophageal phases of swallow [39]. It is also well-reported that esophageal afferents modulate the esophageal phase of swallow, and in general, rapid esophageal distension (EDist) by solid bolus, air bolus, or balloon inflation makes the esophageal phase of swallow more powerful and prolonged [39–43]. However, less is known about the effect of rapid esophageal distension on the pharyngeal phase of swallow, especially how it may alter diaphragm activity. Such effects would have the potential to induce or modulate subsequent/repetitive pharyngeal swallow in response to a bolus in the esophagus.

Several distinct reflexes that result from distension of the upper portion of the esophagus have been thoroughly described by Shaker’s group [41, 44–47]. The authors have divided these reflexes into two main sets: those that are activated by slow distension, and those that are activated by rapid distension. Slow esophageal distension activates the UES and esophageal peristalsis; these reflexes are mediated by muscular tension receptors. Rapid esophageal distension relaxes the UES, stimulates laryngeal adductor and elevator muscles, and stimulates some esophageal contractions; these reflexes are mediated by rapidly adapting muscosal touch receptors [39–41, 45, 48, 49] and have previously been categorized as belch and its component reflexes. These reported reflexes clearly indicate that esophageal sensory input can affect muscles involved in the pharyngeal phase of swallow, but these studies did not aim to specifically test the pharyngeal phase of swallow itself. Esophageal afferent information travels via the vagus nerve to the nucleus tractus solitarius (NTS) in the brainstem, where interneurons (some of which are premotor neurons) influence other esophageal or non-esophageal neurons involved in swallow. The esophageal motor nuclei are nearby in the nucleus ambiguus (NA) and the dorsal motor nucleus of the vagus.

Disorders of the pharyngoesophageal segment include esophageal web, cricopharyngeal bar, and generalized narrowing [50]. Different bolus size and viscosity change the distension required to move the bolus from the pharynx into the esophagus. While these disorders have been well-described, their mechanistic effect on the activation of swallow and the alteration of subsequent swallows in a series is not known. The current study tested the hypothesis that activation of esophageal mechanoreceptors by rapid distension modulates the pharyngeal phase of swallow. This allows for direct comparison of the effects of esophageal distension, water infusion, and the combination of distension and water infusion on upper airway and diaphragm EMG activity during swallow.

## 2. Methods

Experiments were performed on 5 spontaneously breathing adult male cats (3.8 ± 0.2 kg, age 1–2 years). The protocol was approved by the University of Louisville Institutional Animal Care and Use Committees (IACUC), in compliance with the National Institutes of Health Guidelines. The animals were initially anesthetized with sodium pentobarbital (35 mg/kg i.v.; Lundbeck, Inc., Deerfield, IL); supplementary doses were given as needed (1–3 mg/kg i.v.). The right femoral artery and vein were cannulated to monitor i.a. blood pressure and administer i.v. fluids, and a tracheostomy was performed. Physiologic levels of end-tidal CO_2_ (4–4.5%; Datax Engstrom, Datax Ohmeda, Inc, Madison, WI), body temperature (36.2 ± 0.7°C; Homeothermic Blanket Control Unit, Harvard Apparatus, Holliston, MA), and arterial blood gas composition (i-STAT1, Abaxis, Union City, CA) were continually monitored and maintained [16]. Arterial blood gas composition was measured once per hour. Mean ± standard deviations for pH (7.4 ± 0.1), base excess (-4.3 ± 3.6 mmol/L), PCO_2_ (30.9 ± 6.1 mmHg), PO_2_ (105 ± 14.5 mmHg), HCO_3_ (20.1 ± 3.4 mmol/L), and lactate (2.1 ± 4.3 mmol/L) were calculated by pooling data across experiments.

Electromyograms (EMGs) were recorded using bipolar insulated fine wire electrodes (A-M Systems stainless steel #791050) according to the technique of Basmajian and Stecko [51]. Eight muscles were used to evaluate swallow: mylohyoid, geniohyoid, thyrohyoid, thyropharyngeus, thyroarytenoid, upper esophageal sphincter (UES), parasternal, and costal diaphragm. The digastric muscles were dissected away from the surface of the mylohyoid and electrodes were placed on the left mylohyoid. A small horizontal incision was made at the rostral end of the right mylohyoid followed by an incision following the midline for approximately 1cm to reveal the geniohyoid underneath. Electrodes were placed 1cm from the caudal insertion of the right geniohyoid muscle. The thyroarytenoid electrodes were inserted through the cricothyroid window into the anterior portion of the left vocal fold, which were visually inspected post-mortem. Rotation of the larynx and pharynx counterclockwise revealed the superior laryngeal nerve, which facilitated placement of the left thyropharyngeus muscle electrodes. The thyropharyngeus is a fan shaped muscle with the smallest portion attached to the thyroid cartilage; electrodes were placed in the ventral, caudal portion of the muscle overlaying thyroid cartilage within 5 mm of the rostral insertion of the muscle. To place the electrodes within the cricopharyngeus muscle, the larynx and pharynx were rotated counterclockwise to reveal the posterior aspect of the larynx. The tissue was palpated for the edge of the cricoid cartilage and electrodes were placed just cranial to the edge of this structure (for a bilateral recording). The left thyrohyoid electrodes were inserted approximately 1 cm rostral to the attachment to the thyroid cartilage. The sternal diaphragm was placed by elevation of the sternum and the electrodes placed along the dorsal surface.

Swallow was defined as quiescence of the UES with overlapping upper airway activity. Esophageal pressure was measured by placing a balloon catheter connected to a pressure transducer. For distension and pressure recordings, a balloon attached to a thin polyethylene catheter (outer diameter 0.5–1.0 mm) attached to a syringe was placed into the upper esophagus through the mouth and attached to a pressure transducer (TA-100, CWE, Inc, Ardmore, PA). At least 1 hour was allowed between placement of the esophageal catheter and start of stimuli trials. Animals were euthanized with an overdose of sodium pentobarbital (3 mg/kg i.v.) until respiratory cessation, followed by 3cc i.v. of saturated potassium chloride until termination of cardiac activity.

### 2.1 Stimulus trials

**Fig 1:**
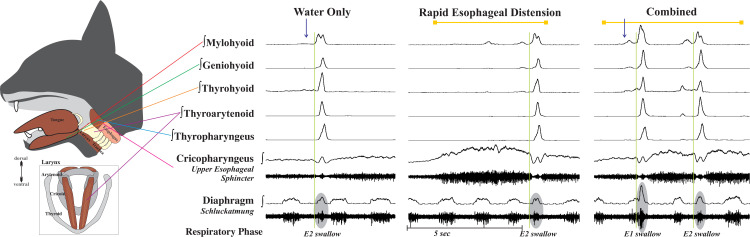
Representative examples of swallow across the three conditions. The combined condition of esophageal distension plus water infusion resulted in a larger EMG amplitude of the thyroarytenoid and diaphragm muscles. Arrows indicate water infusion in the oropharynx, line indicates esophageal distension, and ovals indicate diaphragm activity during swallow (i.e. schluckatmung). Of note, the first swallow in the combined condition has a swallow occurring in the transition from inspiration to expiration (E1 and/or post-I); all others are during late expiration (E2). Muscle EMGs are displayed as integrated traces, but the cricopharyngeus (UES) and diaphragm display raw EMG traces as well. *We hypothesize that the small activity during the UES relaxation is inferior pharyngeal constrictor activity, as the UES in the cat is relatively short.

Esophageal mechanoreceptor activation was produced by rapidly inflating the esophageal balloon with 3cc of air in less than 1 second, then maintaining this pressure for 5 seconds. Swallow was induced by infusing 3cc of water into the oropharynx via 1-inch-long thin polyethylene catheter (outer diameter 0.5–1.0 mm) placed at the back of the tongue (rostral to the faucial pillars). Each animal was subjected to three different stimulus conditions with at least 1 minute between each trial: 1) water only; 2) esophageal distension (EDist) only; and 3) combination: the esophagus was distended by balloon inflation for 5 seconds, and water was infused at the 2.5 second mark. Fig 1 displays representative swallows during each condition.

### 2.2 Data processing and statistical analysis

EMGs were recorded and analyzed using Spike 2 Version 7 (Cambridge Electronic Design, United Kingdom). Moving averages of EMGs were integrated with a 20 ms time constant (Fig 1). Durations were measured as the time between the onset and the point where the signal returned to baseline (ms). EMG amplitude measures were normalized to the largest swallow and are presented as percent of maximum. Pressure transducers were calibrated prior to each experiment, and are presented here as recorded. For all figures, waveforms were exported to CorelDRAW 2020 (v22.1.1.523).

To assess swallow-breathing coordination, a Wilcoxon Signed Rank Test was used. An assigned coding system was used for the breathing phase in which the swallow occurred: inspiration (I; start to peak diaphragm activity) as “1”; early expiration (Yield [52] or E1; peak to end diaphragm activity) as “2”; and mid/late-expiration (E2; end of diaphragm activity to start of next breath diaphragm activity) as “3”. For all tests a difference was considered significant if the *p*-value was less than or equal to 0.05.

**Fig 2:**
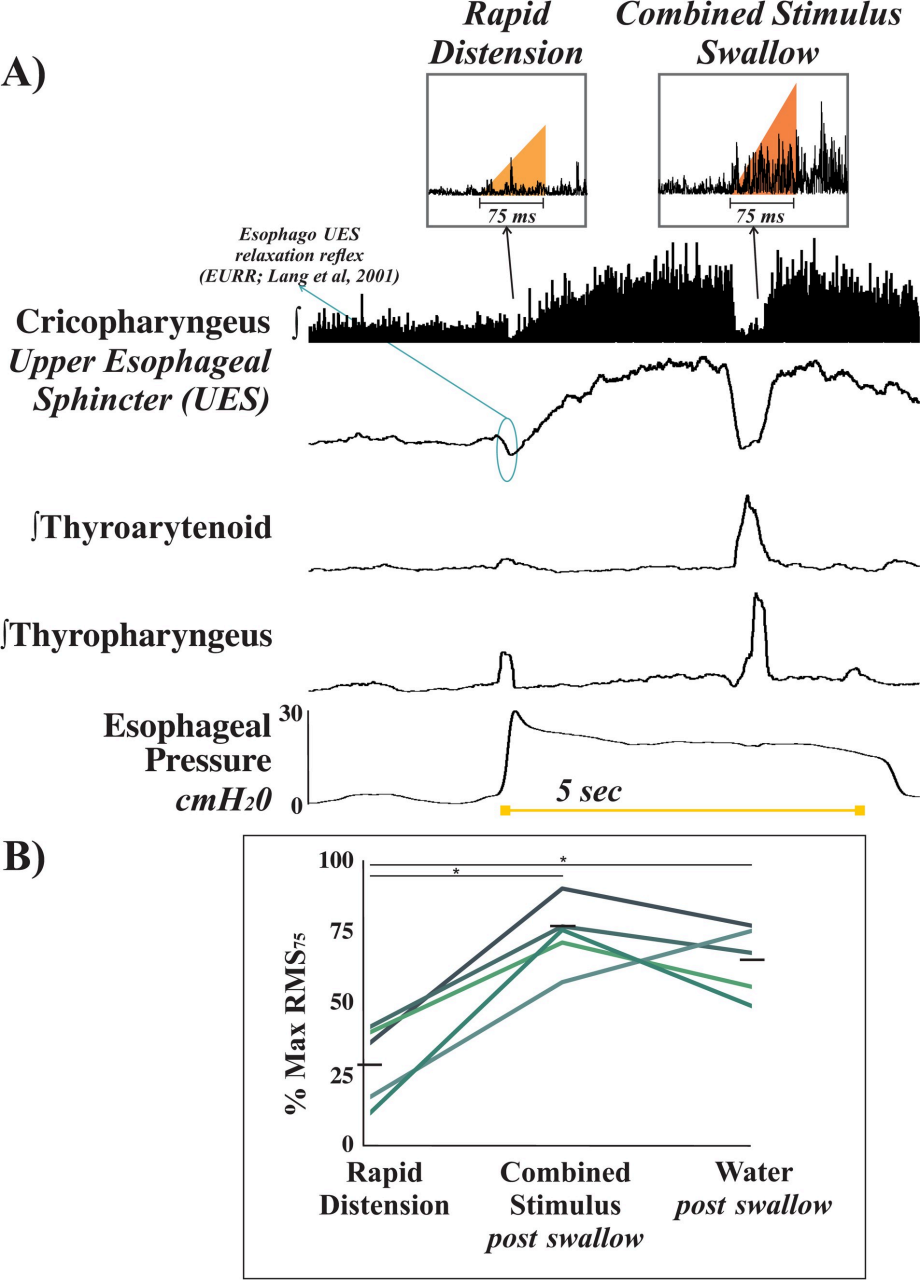
RMS_75_ analysis of upper esophageal sphincter (UES; cricopharyngeus) recruitment. A) Representative example of EMG activity and esophageal pressure during a combined stimulus trial. The root mean square calculation over 75ms (RMS_75_) represents motor unit recruitment of the UES after swallow. The triangles highlight integrated cricopharyngeus activity during rapid distension and post-swallow activity with a combined stimulus over 75ms. Oval highlights a esophago-UES relaxation reflex which is common with rapid esophageal distension. EMGs are displayed as integrated signals with the cricopharyngeus also displaying a rectified raw trace. B) Displays a line graph of individual animal’s change in percent of maximum RMS_75_ across the three conditions, and the black horizontal lines display the group means. *There was a significant increase in UES recruitment during the combined and water conditions compared to rapid distension alone (*p* < 0.05).

A mean ± standard deviation (SD) was calculated for each animal, and then averaged for each condition across animals (Table 1). Student t-tests or ANOVA were performed when appropriate. Pearson’s product moment correlations (*r*) were calculated comparing all amplitude and duration measures to determine relationships between the dependent variables (Table 2). Additionally, root mean square (RMS), a measurement of motor unit recruitment, was calculated using the following transfer equation: $Vrms=\sqrt{AVG}({Vemg}^{2})$, where *Vrms* is the voltage input of the EMG signal and *AVG* is the averaging time constant (75ms), as described by Sieck and Fournier [53] (Fig 2).

**Table 1:** Means, standard deviation (SD), and *p-*values for swallow parameters during conditions of water infusion (W), esophageal distension (EDist), and combined stimuli (CS: W + EDist).

	**Water (W)**			**Esophageal Distention (Edist)**			**Combined Stimuli (CS)**			p-value*		
**Amplitude (% max)**	**Mean**	±	**SD**	**Mean**	**±**	**SD**	**Mean**	**±**	**SD**	**Edist vs W**	**Edist vs CS**	**W vs CS**
**Hyoid/Laryngeal Elevators** Mylohyoid	78	±	11	62	±	5	76	±	11	**0.02**	*0*.*06*	0.8
Geniohyoid	69	±	19	40	±	17	75	±	9	**0.04**	**0.002**	0.5
Thyrohyoid	77	±	8	65	±	16	76	±	5	*0*.*07*	0.2	0.8
**Pharyngeal** Thyropharyngeus	74	±	8	60	±	19	72	±	15	0.2	0.2	0.8
**Laryngeal Adductor** Thyroarytenoid	61	±	21	52	±	11	73	±	17	0.3	**0.03**	0.4
**Schluckatmung** Diaphragm	51	±	18	41	±	10	64	±	7	0.1	**0.01**	0.2
Cricopharyngeus (post-swallow UES)	55	±	20	78	±	12	80	±	7	**0.02**	0.4	**0.02**
	**Water**			**Esophageal Distention**			**Combined Stimuli**					
**Duration (ms)**	**Mean**	**±**	**SD**	**Mean**	**±**	**SD**	**Mean**	**±**	**SD**			
Mylohyoid	405	±	135	348	±	123	372	±	91	**0.02**	0.5	0.4
Geniohyoid	424	±	146	340	±	139	415	±	158	*0*.*08*	**0.03**	0.8
Thyrohyoid	433	±	256	315	±	200	360	±	69	0.5	0.6	0.6
Thyropharyngeus	331	±	87	297	±	46	402	±	118	0.4	0.1	**0.02**
Thyroarytenoid	326	±	35	263	±	53	412	±	67	0.2	**0.02**	**0.05**
Diaphragm	307	±	72	246	±	48	287	±	54	0.2	0.4	0.7
Cricopharyngeus (UES relaxation)	556	±	138	576	±	138	605	±	135	0.8	0.6	0.4
Total Swallow Time	522	±	192	482	±	151	590	±	125	0.5	*0*.*09*	0.3
Laryngeal Elevation Time	464	±	176	397	±	173	453	±	179	**0.007**	0.2	0.8

*p-value < 0.05 in bold.

*p-value approaching significance in italics.

Amplitude is normalized to maximum of control and shown as a percentage. Reported *p-*values are from ANOVA and significant post-hoc tests. Significance is bolded at *p-*values < 0.05.

**Table 2:** Pearson correlations comparing EMG amplitudes and durations during swallow with all data pooled across the three conditions.

		Amplitude							Duration						
		MyHy	GeHy	ThHy	ThPh	ThAr	Dia	UES	MyHy	GeHy	ThHy	ThPh	ThAr	Dia	UES
**Amplitude**															
Hyolaryngeal Elevators	**MyHy**		**0.6**	0.3	*0*.*5*	*0*.*4*	**0.7**	0.04	0.04	**0.6**	**0.6**	**0.6**	*0*.*5*	**-0.6**	*-0*.*5*
	**GeHy**			**0.7**	**0.7**	**0.7**	**0.6**	0.05	-0.2	**0.6**	**0.7**	**0.6**	**0.6**	**-0.6**	*-0*.*5*
	**ThHy**				*0*.*4*	*0*.*4*	*0*.*4*	-0.2	-0.1	*0*.*4*	*0*.*4*	*0*.*3*	*0*.*3*	**-0.6**	*-0*.*4*
Pharyngeal	**ThPh**					**0.6**	0.2	0.01	0.1	0.4	**0.6**	*0*.*5*	0.3	*-0*.*5*	*-0*.*4*
Laryngeal	**ThAr**						0.3	-0.04	-0.3	0.2	*0*.*5*	0.2	**0.6**	**-0.6**	-0.1
	**Dia**							*0*.*4*	*-0*.*4*	0.3	*0*.*4*	0.3	**0.6**	-0.3	-0.2
	**UES**								**-0.6**	*-0*.*4*	-0.2	-0.2	0.3	*0*.*4*	**0.6**
**Duration**															
Hyolaryngeal Elevators	**MyHy**									*0*.*5*	-0.01	0.1	**-0.7**	0.02	**-0.6**
	**GeHy**		0.0–0.2		Negligible						**0.7**	**0.7**	0.2	*-0*.*4*	***-0*.*9***
	**ThHy**		0.2–0.4		Weak							***0*.*9***	**0.6**	**-0.6**	**-0.7**
Pharyngeal	**ThPh**		0.4–0.6		*Moderate*								*0*.*4*	-0.3	**-0.7**
Laryngeal	**ThAr**		0.6–0.8		**Strong**									*-0*.*5*	-0.07
	**Dia**		0.8–1.0		***Very Strong***										**0.6**
	**UES**														

*All data was pooled over the three conditions.

Box = Correlation of muscle amplitude to its duration.

Amplitude is normalized to maximum of control and shown as a percentage. Reported *p-*values are from ANOVA and significant post-hoc tests. Significance is bolded at *p-*values < 0.05. (MyHy = mylohyoid; GeHy = geniohyoid; ThHy = thyrohyoid; ThPh = thyropharyngeus; ThAr = thyroarytenoid; Dia = diaphragm; and UES = upper esophageal sphincter).

## 3. Results

Fig 1 illustrates anatomical placements of the recorded EMGs as well as example traces of swallows produced from each stimulus condition. The representative EMGs are aligned with the rostral-caudal direction of bolus flow. Respiratory cycles are displayed before and after each trial and the respiratory phase of each swallow is noted at the bottom of the figure. Although portions of the thyropharyngeus and cricopharyngeus muscles both participate as part of the inferior pharyngeal constrictor and UES, we placed the electrodes for the thyropharyngeus to be representative of the inferior pharyngeal constrictor and the cricopharyngeus to be representative of the UES activity.

Each stimulus (water, EDist, and combined stimulus) was effective in eliciting swallow. An average of 12.2 ± 3.4 stimuli were administered per animal. An average of 20.2 ± 7.3 total swallows were elicited per animal. Across all conditions, 85% (52/61) of swallows occurred during expiration; 3% (2/61) occurred during inspiration; 3% (2/61) occurred during the transition from expiration-inspiration; and 8% (5/61) occurred during the transition from inspiration-expiration. There were no significant changes in swallow-breathing coordination across conditions.

Table 1 summarizes EMG amplitude (percent of maximum) and duration (ms) means ± SD for each muscle and condition, and results of the statistical comparisons. There were increases in EMG amplitude (percent of maximum) during water infusion compared to rapid EDist in the mylohyoid (26%), geniohyoid (73%), and thyrohyoid (18%, approaching significance), and a significant decrease in UES amplitude (29%). There were increases in EMG amplitude (percent of maximum) during combined stimulus trials compared to rapid EDist in the mylohyoid (23%, approaching significance), geniohyoid (88%), thyroarytenoid (40%), and the diaphragm (56%). Combined stimulus trials also significantly increased UES activity compared to water infusion by 45%.

There were increases in burst duration during water infusion compared to rapid EDist in the mylohyoid (16%) and geniohyoid (25%; approaching significance), and an increase in laryngeal elevation time by 17%. There was an increase in burst duration during combined stimulus trials compared to rapid EDist in the geniohyoid (22%) and thyroarytenoid (57%), and an increase in total swallow time by 22% (approaching significance). Combined stimulus trials also increased thyropharyngeus duration by 21% and increased thyroarytenoid duration by 26% compared to water infusion.

Fig 2 illustrates RMS_75_ analysis of UES activity for rapid esophageal distension and during a combined stimulation trial (Fig 2A), and relative change in RMS_75_ across conditions in the five animals (Fig 2B). The recording in the figure displays an esophago-UES relaxation reflex, but this was not evoked by all stimuli or in all animals. It can also appear to resemble a very small swallow with activity of thyroarytenoid and thyropharyngeus muscles. There was a significant effect of condition on the RMS_75_ of the UES activity [*F*_(2,12)_ = 17.248, *p* < 0.001]; post-hoc testing revealed that the combined stimuli produced larger EMG recruitment than distension alone (Fig 2B) and post-swallow activity in response to water (*p* = 0.001; *p* < 0.001, respectively).

Table 2 is a matrix showing all Pearson Product Moment Correlations for EMG amplitude and duration measures. Due to the relatively small amplitude and short duration of the swallows induced by esophageal distension, there were stronger correlations between EMG amplitude and duration than those reported in our previous publications [13, 17, 38, 54].

## 4. Discussion

Upper esophageal afferent feedback is an important factor in ongoing airway protection risk assessment. Our results confirm that rapid distension of the cervical esophagus (EDist) produces swallow, as shown by Lang, et al. [45], but also demonstrate that swallows induced by EDist have significantly reduced hyoid/laryngeal elevator EMG amplitude and duration when compared to swallows induced by oropharyngeal water stimulation, and shorter laryngeal elevation time (Fig 1; Table 1). Additionally, when the conditions of rapid EDist and water infusion were combined, the thyroarytenoid and diaphragm (schluckatmung) EMG activity increased and laryngeal closure time increased.

The muscular makeup of the esophagus varies by species. The esophagus in dogs, rodents, and sheep is composed entirely of striated muscle, but in cats and primates, the upper (proximal) portion of the esophagus is striated and controlled by cranial motor neurons, and the lower (distal) portion is smooth and controlled by the autonomic system [3, 55]. In humans, the striated portion comprises the upper one-third of the esophagus, which transitions to incorporate more smooth muscle fibers, with the lower two-thirds consisting of entirely smooth muscle [56]. In cats, the upper two-thirds is striated [39]. The striated portion is innervated by motor neurons from the nucleus ambiguus (NA), while the smooth portion by is innervated by autonomic preganglionic neurons from the dorsal motor nucleus of the vagus that synapse with postganglionic motor neurons in the esophageal myenteric plexus [57, 58]. Unlike the oropharyngeal phase of swallow, the esophageal phase is not an all-or-none activity, suggesting a difference in underlying central mechanisms.

Esophageal receptors have been extensively studied for secondary peristalsis (esophageal contraction that is experimentally induced in the absence of the oropharyngeal phase of swallow) [39, 41–43, 45]. In the absence of swallow, activation of esophageal afferents alone stimulates esophageal secondary peristalsis; all esophageal peristalsis is secondary to esophageal stimulation and may therefore require at least a small esophageal bolus [59–61]. When initiated from the upper (striated) portion of the esophagus, secondary peristalsis is controlled centrally, as evidenced by the fact that thoracic vagotomy (to sever afferents but preserve motor efferents to this portion) eliminates the reflex [47]. When initiated from the lower (smooth) portion of the esophagus, secondary peristalsis is controlled peripherally, as demonstrated by the fact that a peristaltic contraction can be evoked in an esophageal smooth muscle segment in the absence of any neural connection with the brainstem [55]. For the primary peristalsis portion of swallow, the pattern in the smooth muscle esophagus is likely dependent on complex interactions between central and peripheral mechanisms [39, 62, 63]. In species with a partial smooth muscle esophagus (including cats and humans), a swallowing wave in the esophagus can alter the subsequent esophageal wave [62], and afferent peripheral feedback during swallow allows esophageal smooth muscle peristaltic contractions to adapt to the size of the bolus [40]. Indeed, swallow produces sequential action potentials in vagal preganglionic efferent [63] that presumably control the smooth muscle portion of the esophagus.

The sensory pathway of EDist-evoked pharyngeal activation is vagal, via the superior laryngeal nerve (SLN), and the recurrent laryngeal nerve caudal to the cricoid cartilage, but not the cervical vagus [41, 58]. There are both rapidly and slowly adapting receptors in the esophageal mucosa [45]. Afferent innervation from these receptors is carried by myelinated A and unmyelinated C type fibers [64, 65]. These fibers are carried by the vagus nerve, project to the nodose ganglion [41, 47, 58], and end in the centralis subdivision of the nucleus tractus solitarius (NTS), which also contains esophageal interneurons, some of which are premotor neurons [47, 66]. Activation of esophageal afferents by balloon inflation in the upper esophagus stimulates discharge of esophageal interneurons in the NTS [67]. Whether any of these esophageal neurons specifically project to oropharyngeal regions is unknown, however, they do converge in the NTS, where sensory information from other regions including the oral, pharyngeal, and laryngeal cavities is pooled and distributed to the swallow pattern generator.

Esophageal stimulation studies that used immunoreactivity of the immediate early gene c-Fos as a marker of neuronal activation showed activity in several brainstem regions, including those known to mediate swallow [68, 69]. Acid perfusion of the upper esophagus, which stimulated belch and/or other pharyngeal responses, activated most of the subnuclei of the NTS, particularly the intermediate, interstitial, and ventrolateral nuclei [68]. Rapid balloon distension of the esophagus stimulated the same reflexes, and activated the same regions, in particular the caudal subnucleus of the NTS [69]. In the cat, these subnuclei are the site of termination of afferents from the trachea [70, 71], and are also the primary pharyngeal premotor nuclei in rats [72, 73]. In contrast, acid perfusion of the lower esophagus, which stimulated secondary peristalsis, activated different subnuclei of the NTS, particularly the central subnucleus [68], as did slow balloon distension [69]. The (pre)motor regions of the dorsal motor nucleus of the vagus and the NA that were activated by the two categories of reflexes also differed. Rapid distension of the esophagus activated NA regions that contain motor neurons for muscles of the pharynx [57, 74–76], larynx [70, 71, 76, 77], and upper airway [74].

Activation of esophageal receptors can stimulate a variety of behaviors including belch in order to prevent reflux of gastric contents, or to create a strong typical swallow and primary peristalsis pattern [39, 41–46, 78]. The main EDist-induced reflexes have been divided into two groups based on their responses to slow or rapid distension of the upper esophagus, although other stimuli may also activate them as well [39]. One distinguishing factor between the groups of slow and rapid EDist-induced reflexes is the activity of the UES; UES relaxation and UES contraction/peristalsis are mediated differently. The cat esophagus contains mucosal rapidly adapting touch receptors [79, 80], and the belch response including UES relaxation is mediated by these receptors [39]. Slowly adapting muscular tension receptors mediate UES contraction and peristalsis. Lidocaine applied to the esophageal mucosa inhibits or blocks UES relaxation, but not contraction [39, 45]. Similarly, capsaicin (which selectively affects mucosal but not muscularis receptors) activates swallowing initially, then desensitizes the swallow response to rapid EDist, raising the threshold required for swallow initiation [39]. When the mucosal layer was completely removed from the esophagus, rapid EDist-induced swallow was blocked, but UES contraction and secondary peristalsis were not [49]. Systemic administration of the GABA_B_ receptor agonist baclofen produced the same results, and also inhibited water-induced swallow and laryngeal adduction [39, 81]. Given these results, rapid EDist must primarily influence the oropharyngeal phase of swallow rather than the esophageal phase. Rapid EDist produces similar reflexes as the EDist-evoked oropharyngeal phase of swallow and accompanying UES relaxation reflex in the current study, therefore we would group these reflexes together.

The pharyngeal swallow pattern generator receives peripheral sensory input from vagal afferents including oropharyngeal receptors, laryngeal receptors, thoracic receptors, pulmonary stretch receptors, esophageal stretch receptors, and possibly thoracic-abdominal receptors [6–7, 17, 25–37]. The swallow sequence is thought to begin first with a synchronized inhibition across all muscles involved, under high peripheral feedback conditions [3, 20, 62, 82–84]. This “deglutitive inhibition” is then removed in a rostrocaudal direction to allow a precise sequential wave of swallow muscle contractions. This activity travels quickly through the oropharynx to arrive at the UES. The esophagus, having also been inhibited at the start of the swallow sequence, remains inhibited during the oropharyngeal stage, but is excited once the oropharyngeal phase is completed. This inhibition of the esophagus involves the brainstem, at least at the onset of the synchronized inhibitory burst, but it may also be mediated by activation of oropharyngeal and/or laryngeal afferents [40]. Indeed, stimulation of the superior laryngeal nerve or inflation of a pharyngeal balloon also inhibit the esophageal stage (likely by a GABA-mediated mechanism) [1, 3, 67, 85].

Studies of repeated rhythmic swallow show that swallows within a bout become stronger across repetitions, both in duration and amplitude. The last swallow in a bout will allow the completion of esophageal peristalsis [3]. While esophageal peristalsis is inhibited during the repetitive swallow bout due to deglutitive inhibition, rhythmic swallowing ultimately facilitates esophageal peristalsis after the last swallow occurs [62]. Peripheral sensory activation decreases the velocity of esophageal peristalsis, making the duration of the whole esophageal phase of swallow longer, and the muscular contraction more powerful [1, 3, 67]. Whether that enhancement is caused by facilitatory or disinhibitory mechanisms is unknown.

Lang, Medda, Shaker, and colleagues [45] found that EDist can induce pharyngeal swallow, and that in general, stronger and more proximal distensions are most likely to activate a pharyngeal swallow response [45]. This was also confirmed in a recent human study of intra-esophageal fluid injections, where swallows were most effectively induced by faster injections, larger fluid volumes, and when the injections were delivered to the upper portion of the esophagus [86]. Interestingly, even with upper esophageal distension there appeared to be no increase in UES tone in these subjects. The present study further confirms that EDist can elicit pharyngeal swallow, and also compares swallow physiology across pharyngeal (water infusion), esophageal (balloon distension), and combined stimulus conditions. Like Shaker’s group [45], we determined activation of pharyngeal swallow through EMG recordings of pharyngeal and hyoid muscles. We also obtained EMG recordings of the diaphragm, which allowed for description of inspiratory muscle activity (i.e. schluckatmung) during EDist-induced swallow. Distinct types of motor units innervate muscles fibers which vary in metabolic and contractile properties. Type I (slow-twitch) fibers produce low voltage signatures and are fatigue resistant, and Type IIB (fast-twitch) fibers are involved in rapid and phasic activity, produce higher voltage signatures, and are prone to fatigue. As force increases, these are recruited in a specific order from smallest to largest (Henneman Size Principle [87]). Studies from Sieck and colleagues [53, 88–90] have used RMS to estimate central drive to the diaphragm, and demonstrate that the recruitment of motor units correlates well with the period of nonstationarity at the onset of the EMG signal. This is usually less than 75 ms, so we also employed the RMS_75_ EMG analysis as a representation of central drive (Fig 2) [89, 90]. The current data support the hypothesis that oropharyngeal stimulation combined with rapid distension increased drive to the upper esophageal sphincter (cricopharyngeus); we believe this reduces airway protection risk by limiting potential reflux.

Our results show that EDist alone elicits a pharyngeal swallow characterized by: decreased amplitude and duration of hyolaryngeal (mylohyoid and geniohyoid) and thyroarytenoid muscle contractions; decreased amplitude of diaphragm EMG; and decreased duration of laryngeal elevation. In contrast, when the swallow stimulus was stronger (water plus EDist: combined stimulation), the schluckatmung (diaphragm EMG) was characteristically ballistic (larger motor units recruited with the potential for larger force production) [91], and the laryngeal adductors produced a longer and stronger contraction. We hypothesize that this functions to protect the glottis from aspiration in the condition of negative intrathoracic pressure created by the increased inspiratory muscle activity. We recently reported that electrical stimulation of the SLN inhibits swallow-related inspiratory activity (schluckatmung) [92], suggesting that SLN afferent feedback may modulate the swallow pattern to protect the airway from an incoming bolus. Combined with our current findings, this suggests that location-specific activation of SLN afferents modulations the swallow motor pattern to increase airway protection during aberrant feeding conditions.

Additionally, we found that hyolaryngeal elevator and pharyngeal muscles were strongly activated as a group. This was evidenced by amplitude correlations to each other, duration correlations to each other, and amplitude and duration correlations with themselves and each other. The amplitude of these muscles was also positively correlated to the amplitude of the laryngeal adductor muscle (thyroarytenoid), and with a more intense schluckatmung (higher amplitude but shorter duration). Also, laryngeal adductor (thyroarytenoid) amplitude was correlated with its own duration. Its duration was also positively correlated with the schluckatmung amplitude, but its amplitude was negatively correlated with schluckatmung duration. When the swallow stimulus was stronger, the schluckatmung (diaphragm EMG amplitude) was larger, and the laryngeal adductors produced a longer and stronger contraction, presumably in order to adequately protect the glottis from aspiration in the condition of negative intrathoracic pressure created by the increased inspiratory muscle activity. Furthermore, the duration of the UES being open during swallow was positively correlated with its own post-swallow contraction amplitude and with the schluckatmung amplitude and duration, but it was negatively correlated with all oropharyngeal EMG amplitudes and durations. Strong schluckatmung activation (amplitude and duration) was correlated with the UES being open longer during the swallow (duration), and with closing more forcefully after swallow (amplitude). These results are consistent with greater activation of oropharyngeal muscles, a more intense schluckatmung, and a longer total swallow duration during stronger swallow stimuli.

This strength of these correlations contrast with our previous publications [13, 17, 38, 54]. This is most likely due to the reductions in swallow amplitude and duration with the esophageal distension stimuli, which increased variability of the dataset, thus revealing these relationships. It is not known if features are inherent to the regulation of the swallow pattern generator or present merely because amplitude and duration were both modified under these conditions. The addition of slow distension trials might also have aided interpretation of these results, and is a limitation of the current study.

## 5. Conclusion

We applied rapid balloon inflation in the cervical esophagus to examine the effects of proximal EDist on pharyngeal swallow physiology. Swallows elicited by EDist alone were characterized by decreased amplitude and duration of hyolaryngeal and thyroarytenoid muscle contractions, and decreased amplitude of diaphragm contraction; in general this swallow was smaller and shorter. This adapted swallow response could function as a clearing mechanism to help prevent aspiration of residual or refluxed esophageal contents. Additionally, swallows elicited by the combined stimuli of both EDist and oral water infusion had stronger diaphragm and post-swallow UES activity, and increased laryngeal closure. Increased schluckatmung associated with these swallows could facilitate superior-inferior bolus propulsion, while increased laryngeal adduction protects against aspiration, and assessment of these features may aid in clinical decisions. These findings implicate brainstem integration of esophageal afferents in the initiation and modulation of pharyngeal swallow.

## Supporting information

S1 Dataset Raw data file. (XLSX)

S1 Checklist Arrive guidelines checklist. (PDF)
